# Resistance and Pseudo-resistance to permethrin: the importance of controlling scabies

**DOI:** 10.3389/fcimb.2023.1297337

**Published:** 2023-11-13

**Authors:** Fabio Rinaldi, Roberta Chirico, Anna Trink, Daniela Pinto

**Affiliations:** HMAP, Human Microbiome Advanced Project, Milan, Italy

**Keywords:** *Sarcoptes scabiei* var. *hominis*, permethrin, pseudoresistance, resistance, scabies

## 1 Introduction

The World Health Organization (WHO) stated that scabies (*Sarcoptes scabiei* var. *hominis*) is one of the most neglected diseases in the world (World Health Organization, 2020). It is estimated that 200 million people in the world have suffered from a scabies infection at least once in their lifetime (Widaty et al., 2022). The update of “European guideline for the management of scabies” is based on the increasing worldwide incidence of this cutaneous disease. Recommended treatments are Permethrin 5% cream applied head to toe and oral Ivermectin 200 μg/kg (Salavastru et al., 2017). Some articles have reported the emergence of *S. scabiei* var. *hominis* resistance to Permethrin. Among the main causes of this problem is treatment failure, which is increasingly documented by physicians and is multifactorial in origin (Lluch-Galcerá et al., 2023). We aim to distinguish pseudo-resistance from true resistance based on the drug’s mechanism of action and mutations. With this letter, we want to underline that we cannot yet talk about true resistance, as there is no statistically significant evidence.

## 2 Permethrin’s mechanism of action

Ion channels are proteins embedded in the cell membrane, creating openings, called “pores”, frequently become the focal points of neurotoxins (Wakeling et al., 2012).. Permethrin acts on the nerve cell membrane by disrupting the function of voltage-gated sodium channels (VGSCs). This results in delayed repolarization and subsequent paralysis and death of the parasite in all stages (Lobo and Wheller, 2021). VGSCs comprise a central subunit that forms the pore (α subunit) and an additional auxiliary subunit. The α subunit contains four domains (I-IV) each with 6 transmembrane segments (S1-S6). The channel pore, responsible for Na+ selectivity, is constituted by the fifth and sixth transmembrane segments (S5 and S6) along with the interconnecting loop. The S4 segments, containing positively charged amino acids, act as voltage sensors, instigating a structural shift that triggers channel opening upon membrane depolarization. Additionally, the cytoplasmic linker between domains III and IV functions as the gate for inactivation. VGSCs can exist in four states controlled by the opening and closing of two distinct “gates,” known as the activation and inactivation gates, respectively. When the membrane is at its resting potential, the channel remains closed, while the inactivation gate is open. Upon depolarization of the membrane, the channel opens, enabling the influx of Na+ into the cell. During the inactivation phase, the inactivation gate shuts, blocking the pore. Subsequently, the channel undergoes closure in a state referred to as deactivation. To return the channel to its “closed” state and ready it for another activation (opening), deactivation must be reversed. Pyrethroids slowed VGSC inactivation and deactivation leading to a prolonged VGSC open time that results in more Na^+^ entering the cell leading to hyperexcitability (Wakeling et al., 2012), delayed repolarization, subsequent paralysis, and death of the parasite.

## 3 Resistance and pseudo-resistance to permethrin

### 3.1 Causes of resistance

One more possible reason for failure treatment is Permethrin resistance which sees its main cause in the occurrence of genetic mutations with the altered drug’s mechanism of action. In recent times, research has pinpointed potential contributors to scabicide resistance, such as VGSCs and glutathione S-transferase (GST). Regarding VGSCs, certain mutations, even outside the drug binding site, can push the channel into a closed state, reducing Permethrin binding. Permethrin tends to bind preferentially when the channel is open or active (Khalil et al., 2017). Pasay and colleagues established a connection between VGSC mutations and resistance in *S. scabiei* var. Canis. They exposed mites to Permethrin over many years, and this resulted in resistance phenomenon. Upon sequencing, a mutation in the α-subunit of the sodium channel was found, distinct from non-resistant dogs in the same population (Pasay et al., 2008). Furthermore, resistance to Permethrin has been associated with heightened activity or expression of GST. This enzyme facilitates drug elimination from the body by catalyzing the formation of a thioester bond between reduced glutathione and Permethrin (Khalil et al., 2017). Although genetic mutations of the mite have been recognized, we cannot yet talk about a true resistance. In a more recent study (Yürekli, 2022), researchers investigated 60 fully mobile mites from patients who exhibited no response to extended Permethrin treatment. Intriguingly, the study showed that Permethrin had a substantial slowing effect on mite movements upon encountering *S. scabiei* var. *hominis*. Moreover, within hours after exposure, all mites were dead, evidencing an absence of resistance to Permethrin. (Yürekli, 2022). While previous studies have investigated Permethrin resistance in mites, it’s worth noting that prior to the research conducted by Yürekli (2022), there had been no specific studies involving mites taken from patients who showed apparent resistance to Permethrin despite extended treatment.

### 3.2 Causes of pseudo-resistance

Some common factors contributing to pseudo-resistance include inadequate counseling by physicians and incorrect treatment, such as administering an insufficient quantity of Permethrin (Cox, 2000); additional factors leading to pseudo-resistance encompass the inadequate duration of treatment (a treatment course that is too short), suboptimal adherence and compliance by patients (Veraldi et al., 2023), and reduced bioavailability of the prescribed treatment. The updated European guidelines for the management of scabies (Salavastru et al., 2017) report that *“Permethrin 5% cream has to be applied head to toe and washed off after 8-12 h of application. This treatment must be repeated after 7-14 days.*” An additional important cause of pseudo-resistance is application errors associated with topical treatment. According to adherence and compliance by patients, an observational study from Nemececk and collaborators (Nemecek et al., 2020) on 21 subjects reported that none of the patients effectively applied the cream following the provided instructions. Consequently, certain areas such as the ankles, interdigital spaces (toes), and sacral region were left untreated. One more reason for pseudo-resistance is that patients stop the treatment when they notice side effects such as irritant contact dermatitis. Additionally, it has been suggested that storing the cream in the refrigerator and applying it as a cool cream could potentially decrease the incidence and severity of this dermatitis (Cox, 2000). Regarding lower bioavailability, considering a pharmacokinetic perspective is essential. The microstructure of the stratum corneum in scabies-affected skin is significantly altered due to inflammation-induced reactive hyperkeratosis and the action of digestive proteolytic enzymes released by the parasites. These enzymes are active in both the mite’s gut and when fecal pellets are released into its environment. As a result, Scholz and colleagues proposed a hypothesis involving hydrophobic interaction between permethrin and keratin (Scholz et al., 2023). Van der Waals interactions between the hydrophobic residues of keratin and permethrin may lead to reduced bioavailability of the latter.

## 4 Discussion

The goal of the management of scabies is to treat patients with appropriate therapy. According to safety and efficacy, the European guideline (Salavastru et al., 2017) proposes Permethrin as the first recommended treatment. In recent years, it was reported an increasing number of cases of persistence of symptoms and signs after treatment (Lluch-Galcerá et al., 2023). It can be due to several causes of resistance (Pasay et al., 2008; Khalil et al., 2017; Yürekli, 2022) and pseudo-resistance (Cox, 2000; Nemecek et al., 2020; Scholz et al., 2023; Veraldi et al., 2023). Our aim is to show that there was no resistance to Permethrin, but the patients’ failure to respond to treatment is attributes to incorrect application of treatment and so to the so-called pseudo-resistance. It’s also necessary family members receive treatment. Successful treatment can also be achieved when physicians choose the most appropriate and specific therapy for each clinical case.

## Author contributions

FR: Supervision, Writing – review & editing. RC: Writing – original draft. AT: Conceptualization. DP: Conceptualization, Writing – original draft.

## References

[B1] Cox N. H. (2000). Permethrin treatment in scabies infestation: importance of the correct formulation. BMJ 320, 37–38. doi: 10.1136/bmj.320.7226.37 10617528 PMC1117314

[B2] Khalil S. Abbas O. Kibbi A. G. Kurban M. (2017). Scabies in the age of increasing drug resistance. PloS Negl. Trop. Dis. :11 (11), e0005920. doi: 10.1371/journal.pntd.0005920 29190303 PMC5708620

[B3] Lluch-Galcerá J. J. Carrascosa J. M. Boada A. (2023). Epidemic scabies: new treatment challenges in an ancient disease. Actas Dermosifiliorg. 132–140. doi: 10.1016/j.ad.2022.07.028 35963332

[B4] Lobo Y. Wheller L. (2021). A narrative review of the roles of topical permethrin and oral ivermectin in the management of infantile scabies. Australas. J. Dermatol. 62 (3), 267–277. doi: 10.1111/ajd.13654 34184244

[B5] Nemecek R. Stockbauer A. Lexa M. Poeppl W. Mooseder G. (2020). Application errors associated with topical treatment of scabies: an observational study. J. Dtsch Dermatol. 18 (6), 554–559. doi: 10.1111/ddg.14122 32469466

[B6] Pasay C. Arlian L. Morgan M. Vyszenski-Moher D. Rose A. Holt D.. (2008). High-resolution melt analysis for the detection of a mutation associated with permethrin resistance in a population of scabies mites. Med. Vet. Entomol 22 (1), 82–88. doi: 10.1111/j.1365-2915.2008.00716.x 18380658

[B7] Salavastru C. M. Chosidow O. Boffa M. J. Janier M. Tiplica G. S. (2017). European guideline for the management of scabies. J. Eur. Acad. Dermatol. Venereol 31 (8), 1248–1253. doi: 10.1111/jdv.14351 28639722

[B8] Scholz L. Fritz C. Chuttke J. Eichner A. Wohlrab J. (2023). Permethrin steal effect by unmasked corneocytic keratin in topical therapy of scabies. Skin Pharmacol. Physiol. 36 (3), 107–116. doi: 10.1159/000529401 36716721

[B9] Veraldi S. Schianchi R. Silvio M. Aromolo I. F. (2023). Pseudoresistance to permethrin in scabies. J. Infect. Dev. Ctries 17 (5), 713–715. doi: 10.3855/jidc.17750 37279414

[B10] Wakeling E. N. Neal A. P. Atchison W. D. (2012). Pyrethroids and their effects on ion channels. Pesticides - Adv. Chem. Bot Pesticides. doi: 10.5772/50330

[B11] Widaty S. Miranda E. Cornain E. F. Rizky L. A. (2022). Scabies: update on treatment and efforts for prevention and control in highly endemic settings. J. Infect. Dev. Ctries 16 (2), 244–251. doi: 10.3855/jidc.15222 35298417

[B12] World Health Organization (2020) Neglected tropical diseases: Treating more than one billion people for the fifth consecutive year. Available at: https://www.who.int/news/item/16-07-2020-neglectedtropical-diseases-treating-more-than-one-billion-people-forthe-fifth-consecutive-year (Accessed 19 January 2020).

[B13] Yürekli A. (2022). Is there a really resistance to scabies treatment with permethrin? *In vitro* killing activity of permethrin on Sarcoptes scabiei from patients with resistant scabies. Dermatol. Ther. 35 (3), e15260. doi: 10.1111/dth.15260 34897912

