# Corrigendum: Resistance and pseudo-resistance to permethrin phenomena: the importance of controlling scabies

**DOI:** 10.3389/fcimb.2023.1358427

**Published:** 2024-01-15

**Authors:** Fabio Rinaldi, Roberta Chirico, Anna Trink, Daniela Pinto

**Affiliations:** HMAP, Human Microbiome Advanced Project, Milan, Italy

**Keywords:** *Sarcoptes scabiei* var. *hominis*, permethrin, pseudoresistance, resistance, scabies

**Text Correction**

In the published article, there was an error. A correction has been made to *Resistance and Pseudo-resistance to Permetrhin*, *Causes of Resistance*, paragraph one. This sentence previously stated:

“Moreover, within hours after exposure, all mites were dead, providing evidence of a genuine resistance to Permethrin.”.

The corrected sentence appears below:

“Moreover, within hours after exposure, all mites were dead, evidencing an absence of resistance to Permethrin.”.

The authors apologize for this error and state that this does not change the scientific conclusions of the article in any way. The original article has been updated.

